# Relationships for Social Change: The Value of the Promotora Framework in Navigating Systems of Employment

**DOI:** 10.1111/jmft.70000

**Published:** 2025-01-24

**Authors:** Danna Abraham, Brian Distelberg, Jan Ewing, Zephon Lister, Lena Lopez‐Bradley, Sandra Ochoa

**Affiliations:** ^1^ California School of Professional Psychology Alliant International University San Diego CA USA; ^2^ Department of Counseling and Family Sciences Loma Linda University Loma Linda CA USA; ^3^ Department of Counseling and School Psychology San Diego State University, CA and the Founder of Narrative Initiatives San Diego San Diego CA USA

**Keywords:** community‐based research, LatinX communities, narrative thematic analysis, promotora framework, socioeconomic mobility

## Abstract

In the United States, socioeconomic disparities are a reality that shapes the challenges many communities of color experience. Throughout the globe, community‐based initiatives have been explored as a way to reduce several barriers that contribute to social inequalities. One in particular, the Promotora framework, has been widely used to improve health outcomes. However, it has yet to be explored to maximize socioeconomic opportunities. The Launch Program, a pre‐COVID‐19 pilot study, aimed to investigate the experiences of LatinX people in one of the first community programs designed to offer a Promotora‐led intervention supporting socioeconomic goals. Researchers collected qualitative data via focus groups from 25 LatinX participants who completed the program. Using narrative thematic analysis, the study explored the potential of a liberatory framework to address employment barriers. Results from this study highlight the benefits of adopting a culturally responsive intervention as a valuable tool in reducing socioeconomic disparities within LatinX populations.

## 1 Introduction

Although the United States is often known as “the land of opportunity,” understanding how to navigate employment systems while obtaining the skills needed for career advancement can be challenging. Employment opportunities directly impact families' financial stability, and nearly 38 million people are currently living below the poverty line (United States Census Bureau 2022). Despite the hopes of some contemporary social policies presented to address this issue, the poverty rate has remained relatively stable at 11.5% of the population since 2021, even following the COVID‐19 pandemic. These statistics raise important questions about conditions in which traditional and new social policies are developed to support opportunities for employment among families.

Given that many public and local programs aim to address social inequalities, the pervasiveness of the high poverty rate among communities of color suggests that these initiatives might not be as impactful as intended. Against this context, it is important to acknowledge that people of color historically have shouldered the disproportionate burden of socioeconomic disparities and its complex implications in their daily lives (Akee, Jones, and Porter 2019; Athreya and Romero 2015).

Historically, race and ethnicity, for example, have been found to be a significant factor limiting fair access to employment opportunities in the United States (Brisson, Roll, and East 2009; McDonald, Lin, and Ao 2009). Furthermore, throughout the literature people of LatinX origin are reported to be three times more likely to live in poverty compared to their European descent counterparts in this country (Denavas‐Walt, Proctor, and Smith 2015; Jones et al. 2016), highlighting the need to broaden our understanding of how these deep‐rooted disparities continue to be a central issue faced by many communities of color.

Several substantially funded programs and social policies continue to focus on offering resources and opportunities to support people economically as primary resources among communities (Athreya and Romero 2015). This trend persisted even during the COVID‐19 pandemic, with the United States government rolling out various financial assistance programs like stimulus checks, enhanced unemployment benefits, rent relief initiatives, and aid for small businesses. However, these policies did not provide equal support to all communities. Undocumented and mixed‐status families, particularly those with undocumented parents, were ineligible for stimulus checks and other forms of federal assistance.

Socioeconomic problems faced by LatinX communities have long been associated with detrimental outcomes such as pervasive health issues (Elias and Paradies 2021), mental health disparities (Villatoro et al. 2018), and limited employment opportunities (Brown 2003), even prior to the COVID‐19 pandemic. Through this, researchers expanded the focus on the impact of social determinants of health to include social and economic factors that influence health outcomes, such as employment, education, and access to resources (Braveman, Egerter, and Williams 2011; Morabia 2020).

Lee, Matthew, and Orpinas (2023) and Wiggins, Borbón, and Young (2013) argue that economic stability interventions – such as those targeting employment – have positive effects on community well‐being and the reduction of disparities in income and access to opportunities. However, there is still a notable gap in exploring how frameworks such as the Promotora address employment and economic challenges, which are vital but often overlooked social determinants of health.

Despite the altruistic intentions of several government assistance program, many researchers argue that current assistance programs overlook contextual variables (Austin, Lemon, and Leer 2005; Nooe and Patterson 2010). For the LatinX community, individual‐level barriers such as language, documentation status, and acculturation are particularly salient. However, it is crucial to also consider broader structural and societal factors. Systemic issues like racism, discrimination, redlining, and other forms of neighborhood and community segregation further limit opportunities for socioeconomic advancement. Galster (2019) and Rothstein (2017) highlight that discriminatory practices, such as housing segregation and unequal access to community resources, can reinforce intergenerational poverty which contributes to additional barriers that limit opportunities for LatinX communities.

One commonly integrated approach explored throughout the literature to address healthcare disparities across several countries is the Community Health Worker (CHW) or Promotora framework (World Health Organization 2020). Central to the Promotora framework is the aim to improve access to quality healthcare services for underserved populations (Ayala et al. 2015; Bush et al. 2014). While this framework has been supportive of underserved communities in healthcare and related fields, its application in domains such as employment systems is undervalued.

For clarity and consistency of language, throughout this paper, we will use the term promotor for male, promotora for female, and promotores for plural including males and females when discussing individual identities or gender‐specific roles. However, when referencing the broader theoretical and practical principles of the framework, we will use the term Promotora framework to align with its widespread use in literature. While this term is female‐gendered, it reflects the origins of this culturally embedded model, which historically emphasized the leadership of women as primary facilitators in the workforce.

The purpose of this study is to understand the experiences of participants who completed a 6‐month Promotora‐led program with a focus on navigating the sociopolitical systems of employment for communities of color. The researchers adopted a qualitative methodology to explore the accounts of participants engaged in the Launch Program – a pilot program with LatinX communities – to understand how relational variations based on the Promotora liberatory framework influenced participants' experiences as they navigated systems of employment within their communities.

## 2 The Promotora as a Liberatory Framework

Frameworks are particularly valuable in addressing complex issues, providing a structured way to organize knowledge by identifying key elements and relationships (Ravitch and Riggan 2017). In this case, we use the term Promotora framework to offer a roadmap emphasizing collaborative processes that center community needs as priorities (Berthold 2016; Pérez and Martinez 2008). For example, Bronfenbrenner's ecological systems theory is well‐known and provides a framework to study human development within interconnected systems (Bronfenbrenner 1979).

Bridging theoretical concepts with practical applications, we acknowledge the power of frameworks as flexible and adaptable while maintaining a structure that guides action. The Promotora framework can be understood as a set of principles that prioritize inclusivity, empowerment, and the promotion of marginalized voices with the hope that everyone has the ability to live well in society (Ingram et al. 2008; Maes 2016). Through the defense of the rights and respect for every individual in society, a liberatory framework acts as a significant instrument in breaking down obstacles to economic advancement and cultivating a fairer and more just living context for more people.

Bush et al. (2014) highlighted that promotores are trusted members of these communities who have deep connections and understanding of the challenges faced by their communities because they experienced them firsthand themselves. However, despite the promising liberatory framework, there is no documentation in the literature discussing the Promotora for the goal of supporting people learning employment systems.

The majority of studies conducted to explore the effects of a promotora‐led intervention with underserved communities have focused specifically on health and behavioral outcomes (Keane, Nielsen, and Dower 2004; Matthew et al. 2017), such as reducing heart health risk behaviors among LatinX communities (Sánchez et al. 2014), improving breast cancer screening practices and completion of cancer treatment among LatinX women (Dudley et al. 2012; Fernández et al. 2009; Livaudais et al. 2010), and improving diabetes‐related behaviors and outcomes among LatinX living in rural areas (Shepherd‐Banigan et al. 2014; Philis‐Tsimikas et al. 2011; Shepherd‐Banigan et al. 2014), to name a few.

While existing research underscores the success of promotora‐led interventions in addressing specific health disparities, extending the framework's application to broader social determinants, such as employment and economic stability, raises methodological challenges. The main critique is the lack of operationalization in training promotores/CHWs highlighting the perceived necessity for standardization to ensure consistency, quality, and accountability in interventions (Lee, Matthew, and Orpinas 2023).

In contrast, there is a robust body of literature that argues against overly manualized and operationalized approaches in community‐based methods, emphasizing the need for flexibility, cultural responsiveness, and context‐specific practices. Researchers like Wallerstein and Duran (2008) and Wiggins, Borbón, and Young (2013) caution against rigid operationalization, highlighting that community‐based participatory research (CBPR) thrives on the adaptability to local contexts and needs. Standardized training risks undermining the organic, reciprocal relationships that promotores develop with their communities, which are crucial for trust‐building and culturally relevant interventions.

Similarly, Bush et al. (2014) argue that the relational aspect of the Promotora framework cannot be effectively captured or taught through manualized training. Lefkowitz (2007) also highlights that promotores often leverage lived experiences, shared identities, and cultural insights to connect with and support their communities. Over‐standardizing training could dilute these relational qualities, replacing them with prescriptive methods that may lack relevance or authenticity in practice.

## 3 The Launch Initiative Program

The Launch Initiative was a promotora‐led program developed pre‐COVID‐19 to assist LatinX families in San Bernardino County in finding better employment opportunities. At that time, LatinX communities were struggling with the lack of employment, a central cause of stress affecting family well‐being. The Launch pilot study emerged from the direct interest of local business community stakeholders.

The Irvine Foundation partnered with local stakeholders – including the Inland Empire Economic Partnership (IEEP) and El Sol Neighborhood Educational Center (El Sol NEC) – to develop and deliver the promotora‐led program in neighborhoods with limited employment opportunities. The IEEP sought the support of the Loma Linda University Lab of Social Policy, Research, and Development to design a research study in collaboration with all stakeholders. This collaboration aligns with the framework principles: involving community members in social activism. One of the key components of this initiative was to include a well‐known community center – El Sol NEC – in the developmental stages of the program. Eligibility criteria for study participants included (1) legal work authorization in the United States and (2) a high school diploma.

Prior to the implementation of the Launch Program, the promotores received a total of 24 h of specialized training throughout a month and 1–2 h of weekly supervision sessions with the guidance of their El Sol NEC supervisor. The training emphasized the development of goal creation, planning, relationship‐building strategies, and participant engagement. These promotores had been actively delivering case management services for several years as part of their job description at El Sol NEC as community partners. All three promotores who delivered the intervention identified as LatinX, with two being women and one a man. Their average age was 57 years, and all reported being married and holding a bachelor's degree. All of the promotores remained in their roles throughout the duration of the study.

Support for participants was individualized based on immediate needs – employment or vocational training – and sessions took place at participants' homes, local community resource centers, or via phone consultations. Promotores collaborated with participants on their employment or vocational goals and provided high‐level support and follow‐up sessions. The intervention tools included structured goal‐setting worksheets, action‐planning templates, and progress‐tracking forms that were used to support participants in setting and achieving their employment or vocational goals. These tools included bilingual handouts and verbal reinforcement during sessions to enhance engagement and comprehension (Bush et al. 2014; Pérez and Martinez 2008).

Distelberg, Carter, and Ochoa (2017) offered an overview of the population who was part of the Launch Program with the average age of participants being 35 years (SD = 12.16), with the sample being predominantly female (67.4%) and all identifying as LatinX. Participants reported having, on average, two children under 18 living at home, as well as one adult child residing in the household. At the end of the program, employment trends were analyzed based on data collected at three time points (i.e., intake, 3‐month mark, and 6‐month completion). From baseline to the 6‐month assessment, full‐time employment among participants increased from 27% to 77%, with a significant decrease in unemployment and part‐time employment.

Beyond employment changes, Distelberg, Carter, and Ochoa (2017) reported a significant finding: a very low attrition rate. Of the 55 families invited to join the study, 46 agreed to participate, resulting in an 84.6% engagement rate. Only four families exited the study during program delivery, yielding a 91.3% retention rate. This retention rate was notably strong compared to similar services for populations facing socioeconomic challenges. The authors attributed the high engagement to the Promotora framework and the strong working alliances developed between participants and promotores.

## 4 Methodology

A community‐engaged research (CEnR) methodology was chosen for this study. CEnR serves as an umbrella for Community‐Based Participatory Research, which has been widely used across the social and behavioral sciences (Ahmed et al. 2015; George 2014). This methodology underscores the importance of engaging with the community to address relevant and meaningful issues, such as socioeconomic challenges (Barkin, Schlundt, and Smith 2013). A distinctive practice and core element of CEnR is the emphasis on partnership between community stakeholders and the research team (Ahmed et al. 2015; Wallerstein and Duran 2008) with the goal of creating reciprocal relationships that support impactful and beneficial research within and across the community.

Community‐engaged research can be a liberatory practice as it emerged in response to traditional research methodologies that often marginalize and overlook the perspectives and needs of the communities being “studied” for the sake of “science.” The methodology closely aligns with the values of the Promotora framework by challenging traditional power structures and research norms, which often favor an expert‐driven approach over community ownership and empowerment. By involving community members in the research process, CEnR has the potential not only to transform the research process but also to shift systems that perpetuate inequalities.

### 4.1 Researcher's Subjectivity Statement

The first author of this study is a woman of color and first‐generation immigrant who transversed language and other barriers as she completed her doctoral studies with a concentration in Marriage and Family Therapy (MFT) in the United States. Fluent in English, Portuguese, and Spanish, she focused on learning Spanish to better support the LatinX community in her role as an MFT, working in both inpatient and outpatient mental health settings. Her language abilities were assets in interviewing, transcribing, and analyzing data, honoring participants' use of preferred language (i.e., Spanish). As a researcher and MFT practitioner, the author is committed to disrupting the order in which training of systemic therapists mostly focuses on apolitical causes of human struggles when conceptualizing and understanding people, problems, and change in clinical and social interventions.

### 4.2 Participant Recruitment

A purposive sampling strategy was employed in this research selecting populations with specific knowledge and experience relevant to the phenomenon of interest (Etikan 2016). To be eligible to participate in the study, individuals had to meet the following criteria: (a) the ability to speak, write, and read in either English or Spanish and (b) current enrollment in the Launch program through the El Sol NEC. The research design was reviewed and approved by the Loma Linda University Human Subjects Review Board (IRB Certificate #5160411).

All of the promotores received IRB training to conduct informed consent with eligible participants. This approach removed the responsibility from external researchers, who may not share a common background with participants, and instead returned agency to the community in the scientific inquiry process.

### 4.3 Participants

From the 46 members who completed the Launch Program, a total of 25 individuals participated in the focus groups. As mentioned in the previous section, 76.9% of the 46 participants finished the program as employed full‐time, which may have contributed to only half of the sample completing the qualitative interviews. The sample who participated in the focus groups consisted entirely of individuals from LatinX backgrounds. A total of 18 females and 7 males were interviewed. The average age of participants was 37 years (SD = 12.19), with ages ranging from 20 to 55 years. As for their relationship status, 41.7% of participants reported being single, 33.3% married, and 12.5% separated from their partner. One hundred percent had a high school diploma or GED.

## 5 Procedures and Analysis

A series of seven focus groups were scheduled to offer participants a range of opportunities to join the study. However, during three of the scheduled focus groups, only one participant attended, which resulted in these sessions being conducted as individual interviews. In total, four focus groups and three individual interviews were conducted with a cohort of 25 program graduates. Before the face‐to‐face interviews, participants were briefed on the study's objectives and provided consent for audio recording. Researchers emphasized participants' autonomy to discontinue their involvement at any time. In consideration of linguistic and cultural contexts, the focus groups were conducted in the participants' preferred language (e.g., English, Spanish, or a combination of both). Each session was approximately 60–90 min long.

The researcher used an interview guide to explore participants' experiences in the program and their relationships with their promotores while also capturing the influence of cultural and contextual factors. Sample questions from the discussion guide included: “Can you speak about the overall experience in receiving assistance from your promotor?,” “What is your employment status, and has it changed while being in the program?.” Participants were also asked to describe their relationship with their promotores, questions included “Can you speak about your relationship with your promotor?,” “Can you speak to the effect of the ethnic background and language in your interactions during the program?.”

The data were fully transcribed by the first author and research assistants. For the interviews conducted in Spanish, the first author was responsible for translating and transcribing the data. To ensure the accuracy of the translations, a bilingual research assistant reviewed both the audio recordings and the transcripts before data analysis. Identifying information was removed, and each participant was assigned a unique identification number to protect confidentiality. ATLAS.ti Scientific Software Development GmbH (2013) was used to assist with the analysis process.

A narrative thematic analysis was used to identify emerging themes within participants' narratives through contrast and comparison methods (Clandinin 2007; Riessman 2007). The first author familiarized herself with the data by repeatedly listening to the interviews. Second, she transcribed and translated the data from Spanish to English. Transcripts were reviewed multiple times, attending to the chronological sequence in the participants' narratives. The first author consulted with an academic reflecting team – who were not involved with data collection or the study. This group composed of bilingual therapists were given the de‐identified transcripts in Spanish and English to assist in the second level of formal coding.

After this step, a group case unit was selected to illustrate the range and variation among the cases that emerged across a thematic map (Riessman 2007). The collaboration between the researcher and research assistants during the transcription and translation of the qualitative data prior to analysis helped address concerns related to rigor in the research process.

## 6 Findings

Our findings suggest that participants found it helpful to be in relationships with promotores, with their identities, with knowledge, and with their families as a way of learning how to navigate employment systems in the United States. The explorations of our findings adopted a framework that invites the reader to consider all learning is relationally and simultaneously happening within and across social interactions.

We explore these four relational components situated in different timelines and aspects of participants' narratives. We bring attention to the expressions some participants used in Spanish and kept them “as is” in their responses based on the understanding that the original language expressions carry nuances that are sometimes lost in translation. Given the extensive nature of the data, we aimed to provide a balance between depth and brevity, and as such, each selected quote represented a key theme or insight that emerged across participants' narratives. This selective approach presents diverse experiences within the group, focusing on the representativeness of individuals' broader experiences central to the study.

Through the connections and familiarity with promotores, participants reported an increased sense of freedom moving through challenging systems of employment. More specifically, community learning was particularly meaningful to how participants described and situated their experiences in the Launch program. To protect participant identities, pseudonyms are used throughout this section. See Table 1 for the following themes that emerged from our narrative analysis.

**Table 1:** Results from thematic analysis.

Themes	Subthemes
Relationship with promotores	a. Shared personal connection to language and cultural similarities with promotores b. Promotores relational ethics c. Honoring the participants' desired destination.
Relationships with identities	a. Employment self‐inventory b. Attending to dominant gender cultural norms c. Accountability towards identified employment goals d. Enhanced agency
Relationship with knowledge	a. Expanding the utilization of community resources b. Applying knowledge into practice
Relationship with family	a. Experiences with family

### 6.1 Theme 1: Relationships With Promotores

Individuals who participated in the Launch program described the importance of developing a relationship with their promotor(a) as well as the significance of sharing personal similarities such as language and ethnicity while working toward their employment or vocational preferences with their promotor(a) – (a) shared personal connection to language and cultural similarities with promotores, (b) promotores relational ethics, and (c) honoring the participants' desired destination.

#### 6.1.1 Subtheme: Shared Personal Connection to Language and Cultural Similarities

Participants described the importance of sharing a personal connection to language related to their preference to express themselves in Spanish as well as their cultural similarities with the promotores.

I think that we (myself and my family) were able to identify a little bit more with my promotora because I'm from the same background. She is Latina, so we could speak both languages (English and Spanish). My promotora could also interact with my mom more so I didn't have to translate. I think that we identified with my promotora most of all because of the language.

(Lucía, female participant)

When discussing the participants' relationships with their promotores, participants stressed the significance of speaking the same language as a supportive component in their experience throughout the program.

I don't speak much English. so for me, it is important to express myself … we have to be on the same page. it would be more difficult and less interesting to be with a promotora that wouldn't understand me so well and I couldn't express myself.

(Isabel, female participant)

While participants described the importance of being able to express themselves in their preferred language, communicating in Spanish was reportedly a contributor to their engagement in the program.

#### 6.1.2 Subtheme: Promotores Relational Ethics

Participants described several aspects of their experience with promotores in terms of building relationships and promotores' unique abilities honor the participants' goals. This theme was identified throughout the participant's responses as an important set of relational ethics when promotores were delivering services.

My promotora's attitude was ample and positive, friendly, open‐minded. This is one of the ways a person will sense you have someone there supporting you. Someone who is by your side, guiding you, and passing on information, such as other ways that one may find the employment they are seeking.

(Mariana, female participant)

When engaging individuals in the program specifically designed to improve socioeconomic mobility, the promotores' practice of relational ethics – centered not only on listening but also on respecting and valuing the relationship with participants – emerged as a crucial factor. This ethical approach involved honoring participants' desires, acknowledging their agency, and fostering mutual trust, which created a foundation for meaningful collaboration.

I think it was important to have her there to listen to me because there are certain things that I already knew (about employment), but I couldn't talk to others – such as my mom for example. Maybe (my mom) wouldn't understand it as much as my promotora would know as she hasn't gone through those types of situations here.

(Camila, female participant)

I opened my mind to my promotor, I told him something personal… and it helped me a lot, I know he counts like a psychologist, I think that maybe something like that… like empathy to work.

(Andrés, male participant)

#### 6.1.3 Subtheme: Honoring the Participants' Desired Destination

In addition to the ways participants described their preferences in speaking the same language and their promotores' ability to join them in their journey of seeking employment, participants shared that in order to help with their employment goals, promotores needed to be sensitive to individual needs and desires in the program.

I believe promotores need to know what are our goals and desires we have to improve, whether it is work if it is family problems, or what reason we haven't found a job. They need to know many things really. When I got connected with him, then we got to talk little by little, what are my desires, if I want to work or study, what are your desires, it is everything that they will know little by little that we will meet every week.

(Santiago, male participant)

It is important for promotores to have this tolerance, to be open because we all have differences in cultures and many differences can happen, but the most important is to focus on what one's doing and reach the goals that one wants to improve.

(Juanita, female participant)

### 6.2 Theme 2: Relationships With Identities

One of the ways participants explained their trajectory in the program was to describe how promotores assisted in broadening preferences in their identities, in the context of navigating the systems of employment. Through their responses, individuals chronologically storied their experiences as they reflected on the changes between the start and completion of their participation in the program. For many of them, promotores assisted in re‐storying their identity preferences and served as a critical force for their employment possibilities. For participants, to re‐imagine this emerging vision of employment goals, they offered insights linked to the road to pursuing socioeconomic mobility while in the program. This process included: (a) employment self‐inventory; (b) attending to dominant gender and cultural norms, (c) accountability toward identified employment goals, and (d) enhanced agency.

#### 6.2.1 Subtheme: Employment Self‐Inventory

Throughout the responses about their experience in the program, participants discussed the trajectory of joining the program while identifying previous employment experiences that are linked to their desire to improve their socioeconomic status.

I was in a job that was very demanding, missing a lot of time with my daughter, now that she is 1 ½ years old, she needs me… So, I felt that I couldn't make changes… I felt stuck where I was.

(Stella, female participant)

Participants discussed their experiences as they joined the Launch Program in relation to the difficulty in finding employment and even applying for government assistance programs to help with income insecurities.

I was facing a very difficult situation too, very difficult emotionally, and I thought I wouldn't get out (sigh) (pause) (tears). ¡Ay!, estoy emocional (I am emotional). I've never imagined myself receiving government assistance, I had to do it because of the situation I was facing, when my salvadora (savior) arrived. I was doing a little work where I was, but I didn't have much work, especially with a child.

(Elena, female participant)

#### 6.2.2 Subtheme: Attending to Dominant Gender and Cultural Norms

As participants progressed in the program being assisted by their promotores, they reflected on gender and cultural norms that influenced their own ideas of employment possibilities. This was particularly true for women participating in the study. Throughout their responses, they provided insight into the shifts in how they related to gender and cultural norms and how it assisted many of them in their journey to seeking better employment opportunities.

I am the oldest of ten children that my mother had, and I had to help my mom take care of them since I was 8 years old, so I didn't have the opportunity to study, not because I didn't want to, but because I couldn't (tears). I came to this country when I was 18 with an 8‐month‐old daughter. I started to go to school and study English, seeking opportunities to overcome challenges.

(Carmen, female participant)

For me, it was very difficult. Emotionally I think I still struggle because I feel alone with my children. When I feel bad, when I feel down, I don't know what to do. It is like walking in circles, even though you know where you want to go, but the children and the family get in the way of the things you want to do to improve and move forward.

(Rosa, female participant)

The women who participated in the study explored the influence of culture and gender while they pursued their employment goals. Their accounts described how they experienced a lack of family support which was connected to gender and cultural expectations while navigating their life with their promotores.

This is our mistake, as Latinas. I was at home and (my family asks) to come here or go there. Sometimes I didn't do things for myself. Why do I have to go here and there (for them)? I wanted to do some things first, like get my diploma, also my GED, now my driver's license, but when I focused on working from home, I spent more time (taking care of) my children.

(Stella, female participant)

The women in the program recounted many times in which they described the difficulties involved as they actively worked toward their goals in the program while still fulfilling gender roles, such as caring for an infant while pursuing career goals.

It is difficult. I don't have anyone to take care of my daughter… it is double the difficulty, I didn't know what to do. I had to take the children to school, I came back to do a little more studying and then to work at night from 11 pm until 6 am in the morning, and everything was very difficult… I wasn't able to sleep for months.

(Luz, female participant)

#### 6.2.3 Subtheme: Enhanced Agency

Overall, participants reported that receiving services from their promotores influenced change in aspects of feeling capable and confident about themselves. In the case of finding employment, participants reflected that their relationships were supportive in becoming agents of change for the purpose of finding employment throughout the program.

I used to not believe in myself, I knew I had potential, but I didn't know how to start… My promotora gave me strategies and I am satisfied with the program, it helped me a lot, and I learned a lot.

(Teresa, female participant)

I have done things that I thought I couldn't do, find a job within 3 months? I thought I couldn't do it. I was feeling very insecure, and I thought I couldn't do it. I was taking a step forward and a thousand steps backward (laughs). Now I feel I can move forward. Things have not been easy because I come from a situation, from a marriage in which my husband controlled everything, financially, what I was eating, what I was wearing, everything.

(Sofia, female participant)

At first, I wasn't going to school, and I wasn't working. I was looking for work, but I wasn't able to land a job anywhere. I lost confidence. (My promotora) helped me get back on track. She helped me saying I could do it. I just keep applying, and I got the job and I've already been working there for 2 months. I went from a job that I didn't really like, to a job that I like, and I want to make it my future career.

(Clara, female participant)

When we seek something we find resources that we didn't know were there… so these difficult moments push you to be stronger, wiser… and gives you more confidence when you start to solve all these things, you know you are much more capable of doing things that you ever thought.

(Ricardo, male participant)

### 6.3 Theme 3: Relationships With Knowledge

Throughout their responses, participants provided insight into the shifts in the ways they related to knowledge and resources available in the community. As they continued to work with the promotores to achieve their employment goals, individuals explained how their relationship with what they learned helped with specific outcomes in their lives. Among them were (a) expanding the utilization of community resources and (b) applying knowledge into practice.

#### 6.3.1 Subtheme: Expanding the Utilization of Community Resources

A significant factor in the process of navigating the systems of employment while in the program was a sense of increased utilization of available resources in the community. Through the Promotora framework, participants expanded the relationship they had with community resources that were already available but most often being underused by community members.

I have two places I can go, the workforce development center. I don't have a computer, I know where to drive to get work done and find ways… because now I have a lot of knowledge, and I know how to do it.

(Marcio, male participant)

#### 6.3.2 Subtheme: Applying Knowledge Into Practice

Participants reported they gained unique knowledge from engaging in the program that was instrumental in taking informed action. The process of gathering knowledge and information relevant to employment goals played an important role in transferring information to practice.

…the most important was that where I started working and applied what I had learned at school and then this was what most helped me to understand what I had learned helped me. It helped me to see the changes. The changes that happened in my job gave me the encouragement to reach for more.

(Teresa, female participant)

I learned the essence and the knowledge de que uno puede (a person can do it)…. in my case, siento que ahora soy yo (I feel that now I am myself). I have the knowledge that I can do it and will do it when the program is over I will take this lesson that I can keep going. (Joelle, female participant)… because I work in the field, in construction, I work hard under the sun every day, what my promotor taught me is that I don't have to settle. There are jobs, if you want to get a career, as contractor, engineer, or architect, this will be what you are going to focus on. I learned to have love and respect for (my) job (that's)what my father has taught me. My promotor has taught me that you can transform your job into a career.

(Mateo, male participant)

I am conscious that I can have the knowledge and all these tools, but if I don't use them. they will just be there. If you are hungry and you to the fridge and say: there is no food and if you don't prepare something, you will die of hunger just looking. Or if you are sick, and you go to the doctor and they give you the best medicine, if you don't take it, nothing will happen.

(Rafael, male participant)

I am studying to get my contractor's license, to expand my skills, and I expect to continue with what I learned with my promotor, he is a good person. Sometimes I would get to the meetings feeling tired, but there was he, with the desire to keep going, and I am very thankful to him.

(Sandro, male participant)

### 6.4 Theme 4: Relationships With Family

The last theme that emerged from our analyses was related to the shifts in relationships with family members reported by the participants of this study. Although promotores did not provide direct services to family members, participants reported a significant difference in the relationships between themselves and family members. In addition, participants identified noticeable changes in the ways they related to family members and vice‐versa. To this end, the subtheme (a) experiences with families, created the path to working toward employment preferences contributed to family transformations throughout participants’ experiences while in the program.

#### 6.4.1 Subtheme: Experiences With Families

The effects of the program extended far beyond the individual participants, reaching into the lives of their families. While promotores may not have direct interactions with participants family members, the transformative changes that participants experience are discussed below in how they influenced the experiences with their families.

All the sessions with (my promotora) definitely made an impact in my life. It has reached my daughters, nieces, parents, siblings… even my sister has said: You can be our own promotora. Directly and physically (my promotora) did not interact with my family… but the impact (exclamation!) she had in my life, and has reached a lot of people. of my family.

(Lia, female participant)

I observe changes in my family. I have a younger daughter, she is 13. She wants to do the same that I have done, to go to the university, to study, and she already told me: Mom, I know you worked so hard, that you don't sleep, that you have done so much work, she said: I am very proud of you. For me, it is the biggest satisfaction, that she is happy and that she at a young age already has this motivation to want to study. She has this dream to go to a university.

(Beatriz, female participant)

My promotora has never had contact with my husband. or my kids, but what she has done to me, (my family) has been noticed. My husband says I am not the same person. He tells me he needs help to clean his credit, things he knows I know. He says before I was a lap cat and I say: I am not the one you think I was. He says now I talk, I help, I work, I travel. I am not weak(anymore).

(Solimar, female participant)

## 7 Discussion

The unique and innovative approach of the Launch program identified individuals from San Bernardino County to step into leadership roles as promotores with the main goal of assisting participants in navigating employment systems. As discussed in the literature, promotores have a unique social location that connects lived experience and local knowledge which have been noticeable in the undoing of structural inequalities in communities of color (Matthew et al. 2017; Pérez and Martinez 2008). Our findings suggest that expanding the Promotora framework for socioeconomic mobility offers a promising path to address socioeconomic inequalities.

The documentation linked to the Promotora framework in healthcare systems inspired the designers of the Launch Program to target socioeconomic challenges in one of the first programs designed for this goal. Using a liberatory framework to build community solutions, our research highlighted the meaningful process of developing relationships between promotores, participants, stakeholders, and researchers. The familiar and relatable nature of these relationships made the quality of the intervention particularly impactful, a finding that aligns with Ingram et al. (2008), emphasizing that community‐based models, particularly those integrating promotores, thrive on the relational trust and shared cultural understanding. During the program, participants reported feeling more supported as they navigated employment system, highlighting the significance of community connections in fostering active participation and empowerment.

The theme of developing relationships aligns with findings from Shepherd‐Banigan et al. (2014), which emphasizes the importance of people bridging connections and sharing personal knowledge. Throughout the accounts of participation in the program, individuals engaged with their promotores shared their newfound personal agency which transformed their ability to seek better employment opportunities. The relationship with promotores helped participants re‐imagine their lives, addressing not only direct goals such as employment needs but also helping participants challenge dominant gender and cultural norms and improve their relationships with family, which led to a newfound sense of agency and confidence in their own lives.

## 8 Implications for Clinical and Community‐Engaged Practices

Despite the promising social justice components of the Promotora framework, the field of behavioral sciences and MFT has not explored its applications for navigating employment systems which are critical for a person's overall social well‐being – especially for communities of color. The existing literature on this framework, even post‐COVID‐19, highlights there is a need for further exploration of its role in supporting communities not only in healthcare settings in alignment with Ingram et al. (2008).

A clear implication for clinical and community practice is the need for the integration of relational models that can be supportive in all systems of human experience (e.g., employment, relational, mental health, and educational). Congruent with Morabia (2020) and Shepherd‐Banigan et al. (2014), community programs that advocate for a collective and socially conscious approach can help build stronger connections across various community systems and also serve as a reminder of the value of interdisciplinary collaborations between fields that already have a history in integrating the Promotora framework.

In addition, there is a need for active advocacy at state, local, and federal levels to expand employment opportunities with living wages. Especially when the community is involved in addressing their own social problems, our findings can strengthen the importance of collective efforts instead of merely suggesting individuals are responsible for coping with inequities in government offices, in therapy rooms, or on the streets.

By advocating for policies that prioritize quality, dignified work opportunities, and relationships at the core of their approaches, people who have shared knowledge (i.e., promotores) can play a vital role in supporting more than healthcare needs. The practical implications of this approach align with a systemic perspective, which can help in the undoing of isolation and nurture community relationships for local and global change. Leveraging this grassroots framework could be a departure from adopting a “white savior” stance that numerous mental health practitioners are encouraged to operate from when supporting communities of color.

## 9 Strengths and Limitations

One key strength of this research is its community‐engaged methodology, which prioritized participant voices and culturally relevant practices that promoted relationships as systemic practice. However, we acknowledge some limitations. First, the reliance on voluntary participation introduces the potential for selection bias, as participants who were more engaged or had more positive experiences were more likely to participate in the interviews. Second, while the qualitative design provides deep insights, the findings may not be generalizable to all LatinX communities. Third, the study was conducted within a specific geographic and cultural context, which may limit its applicability to other regions or demographic groups. Finally, because our focus explored the overall experiences of participants in the program, we may have overlooked the unique aspects that could connect participant's narratives and their background, particularly in terms of their family dynamics, as our data collection prioritized capturing collective experiences rather than individual‐level details.

While the limitations discussed above focus primarily on the methodology and scope of the study, it is also important to consider broader state and federal policies that potentially affect LatinX communities, such as eligibility for financial assistance or work authorization. Policies that restrict access to resources for undocumented or mixed‐status families may have exacerbated challenges for some participants and could have influenced the program's overall impact on socioeconomic mobility and mental health outcomes.

## 10 Conclusion

For mental health and systemic health professionals, our findings stress the importance of collaboration and interdisciplinary approaches to social problems. By integrating an understanding of systemic and structural factors affecting people of color, professionals are invited to bridge the gaps between mental health and broader socioeconomic challenges faced by many people at the margins of society. Embracing the Promotora framework to enhance socioeconomic shifts presents a valuable opportunity for empowering community advocacy and promoting societal welfare. Although this research offered valuable contributions using an interdisciplinary approach, we recognize inherent limitations. We have described our efforts to conduct culturally sensitive research by inviting a reflecting team in the process of coding the data.

The connections individuals build with resources and supportive networks were found to be central to achieving better employment opportunities. Relationships play a powerful role in this process, as they provide the advocacy and knowledge bridging necessary to overcome barriers. The Promotora framework aligns with systems thinking, where MFTs can approach the role of knowledge brokers (i.e., promotores) to help people navigate complex challenges and connect with the resources they need. While the program primarily focused on employment, its impact extended far beyond job placement.

Participants not only developed a stronger sense of agency in their professional lives but also gained a renewed sense of self‐worth and strengthened family dynamics. These ripple effects demonstrate how empowering individuals through support systems not only enhance their confidence in achieving employment goals but also fosters broader personal and relational development, ultimately leading to more sustainable success.

Overall, the findings from our study highlight the potential for community‐based frameworks like Promotora to foster lasting change within communities of color. As we look into the future, the results offer valuable insights for community‐engaged mental health practitioners, suggesting that the Promotora framework could be a useful tool in refining both the theoretical and practical applications of systems thinking.

Given its positive impact on employment, self‐agency, and family dynamics, further exploration is needed to expand the role of MFT practitioners in the implementation of Promotora‐led programs. Future research should consider adapting this model to different populations and contexts – both within the United States and globally – to fully understand its potential to address systemic inequalities and empower diverse communities.

## Disclosure

This study was presented at the 2019 American Family Therapy Association yearly Conference in Oakland, California, and the 2019 National Conflict Family Resolution yearly Conference in Fort Worth, Texas.
